# Application of sticky bone combined with concentrated growth factor (CGF) for horizontal alveolar ridge augmentation of anterior teeth: a randomized controlled clinical study

**DOI:** 10.1186/s12903-024-04229-2

**Published:** 2024-04-08

**Authors:** Yu Xie, Yanyan Qin, Miao Wei, Wenzhi Niu

**Affiliations:** 1 School of Stomatology, Xuzhou Medical University, Xuzhou, Jiangsu China; 2 Department of Implant Dentistry, Affiliated Stomatological Hospital of Xuzhou Medical University, Xuzhou, Jiangsu China

**Keywords:** Alveolar ridge augmentation, Sticky bone, Concentrated growth factor (CGF), Guided bone regeneration (GBR)

## Abstract

**Objective:**

This study was designed to estimate the effect of sticky bone combined with concentrated growth factor (CGF) on anterior alveolar horizontal augmentation during implantation.

**Methods:**

Twenty-eight patients were randomly assigned to either the test group (Group 1, *n* = 14) or the control group (Group 2, *n* = 14). Patients in Group 1 and Group 2 underwent GBR using sticky bone combined with CGF and bone powders mixed with saline, respectively. On postoperative Day 7, the patients completed the visual analogue scale (VAS). Three-dimensional models of maxillary alveolar bone were reconstructed from CBCT data at different periods, and the bone volume conversion rate was calculated with the assistance of a measurement marker guide. Labial bone thickness before and after trauma closure and bone density at six months postoperatively were also measured.

**Results:**

The mean bone volume conversion rate for Group 1 (72.09 ± 12.18%) was greater than that for Group 2 (57.47 ± 9.62%, *P* = 0.002). The VAS score was lower for Group 1 than for Group 2 (*P* = 0.032). At six months postoperatively, greater bone density was found in patients in Group 1 than in those in Group 2, although the difference was not statistically significant (*P* > 0.05). The change in the thickness of the labial bone graft material in Group 1 was smaller than that in Group 2 (*P* = 0.025).

**Conclusion:**

Sticky bone combined with CGF was able to achieve better bone augmentation than conventional GBR. With excellent mechanical properties and the capacity to release growth factors, sticky bone is an ideal material for bone grafting.

**Trial registration:**

The study was registered at the Chinese Clinical Trial Registry on 10/04/2022 (Identification number: ChiCTR2200058500).

## Introduction

Advances in digital technology and biomaterials have transformed traditional implantology, improving precision, efficiency, and patient outcomes [1, 2]. In the oral implant field, the implant restoration of missing teeth in the aesthetic region is of great concern to both patients and doctors [3]. However, the labial bone thickness of nearly 90% of patients with missing maxillary anterior teeth is less than 2 mm [4]. It was found that 86.2% of patients with dental implants required bone augmentation techniques to achieve more than 1.5–2 mm of bone thickness on the labial surface of the implant to maintain the long-term stability of the implant and soft tissue [5, 6].

Guided bone regeneration (GBR) employing bone substitute material and a resorbable collagen membranes is currently a common clinical technique for bone augmentation [7–10]. Conventional GBR, however, has several disadvantages, such as a lack of rigidity and the tendency of the bone graft to displace [11], which may result in the resorption of the enhanced alveolar bone [12]. Soft tissue pressure during surgical incision closure and the healing process can accelerate the collapse of grafts. Therefore, how to improve the spatial maintenance ability of graft materials is the focus of GBR-related research [13, 14].

Recently, autologous plasma products have become increasingly sophisticated in the field of oral regeneration. Compared with platelet-rich plasma (PRP) and platelet-rich fibrin (PRF), third-generation concentrated growth factor (CGF) utilizes variable-speed centrifugation to obtain a denser, more growth factor-rich fibrin matrix [15, 16]. According to Sohn et al. [17], mixing autologous fibrin gel (AFG) prepared at the same time as CGF with bone powder particles can produce a bone graft matrix rich in growth factors (sticky bone).

Sticky bone requires no biochemical additives, is quick and easy to prepare, and can be moulded into different shapes to fit different bone defects. Moreover, the volume and contour of the bone powder particles can be maintained during the healing period. Sohn et al. [17] reported that the use of CGF and sticky bone in the restoration of one- and two-wall bone defects and immediate implantation can lead to better clinical results. A retrospective study by Barbu et al. [18] reported that the clinical results of horizontal bone enhancement were comparable whether the bone-shell technique or GBR with CGF-enriched bone graft matrix (sticky bone) was used. All of these methods can achieve a sufficient increase in bone width to allow for implant placement in the desired location. A randomized clinical trial by Tony et al. [19] demonstrated that sticky bone could achieve desirable horizontal bone width increases even without the use of collagen membranes. All of the above studies suggest that sticky bone is a promising biomaterial that deserves more attention.

Most of the current studies concerning the effect of alveolar ridge horizontal increments have used a two-dimensional measurement method, in which the horizontal width of the alveolar bone at several baselines is measured via CBCT. In contrast, the three-dimensional calculation of volumetric changes has more applications in the assessment of maxillary sinus augmentation results and is more accurate than linear measurements [20]. However, this method is rarely employed because there is often no clear contour range when the alveolar ridge is locally augmented. To address this point, we designed measurement marker guides to provide reference planes to limit the uniform range of the maxillary model in different periods, enabling accurate measurement and calculation of changes in bone volume.

Although autologous plasma has been widely used in oral regeneration, there is still a lack of clinical studies on the combined application of sticky bone and CGF. Therefore, in this study, we aimed to use an innovative three-dimensional volumetric calculation method to compare the outcomes of horizontal bone augmentation utilizing sticky bone in combination with CGF in the anterior maxilla with those of conventional GBR. The hypothesis of this study was that the combination of sticky bone and CGF would be significantly superior to conventional GBR in terms of the bone conversion rate, visual analogue scale (VAS) score, density of newly formed bone, and change in thickness of the labial bone graft material.

## Method

### Study population

This was a single-centre, prospective, controlled, randomized clinical trial. The study was registered at the Chinese Clinical Trial Registry on 10/04/2022 (Identification number: ChiCTR2200058500). The subjects were recruited from the Department of Implant Dentistry, Affiliated Stomatological Hospital of Xuzhou Medical University from June 2022 until December 2022. Written informed consent was obtained from the patients. The study protocol was approved by the ethical committee of the Affiliated Stomatological Hospital of Xuzhou Medical University (Approval number: 2022-KY-005-02).

#### Inclusion criteria

1. Patients aged 18 to 60 years old (including those aged 18 years old and 60 years old).
2. The presence of a single anterior tooth missing with a horizontal bone defect and suitable for implantation-concurrent bone augmentation (alveolar ridge class II, II, and IV with a width of at least 4 mm according to the classification of Tolstunov et al. [21])
3. Good oral hygiene control.
4. The doctor’s orders were followed, and the patient returned on time.

#### Exclusion criteria

1. Heavy smokers (≥ 10 cigarettes per day).
2. Local or systemic contraindications for implant surgery.
3. History of previous alveolar ridge preservation.
4. Uncontrolled periodontitis.

### Sample size calculation and randomization

Based on the results of the preexperiment (mean 1 = 0.72, mean 2 = 0.57, S1 = S2 = 0.1), the sample size was calculated in PASS 15 as 22 using an alpha level of 5% and a beta level of 20%, that is, 80% power. Referring to similar studies and taking into account a 30% dropout rate, 28 subjects were ultimately enrolled in the study. The patients were randomly allocated to 2 groups using the random number code table method. Fourteen patients were included in the test group (Group 1), in which sticky bone combined with CGF was used for bone augmentation, and the other patients were enrolled in the control group (Group 2), in which bone powder mixed with saline was used for bone augmentation. The evaluator who was in charge of the assessment of the CBCT measurements was blinded in this study.

### Surgical procedures

**Fig. 1:**
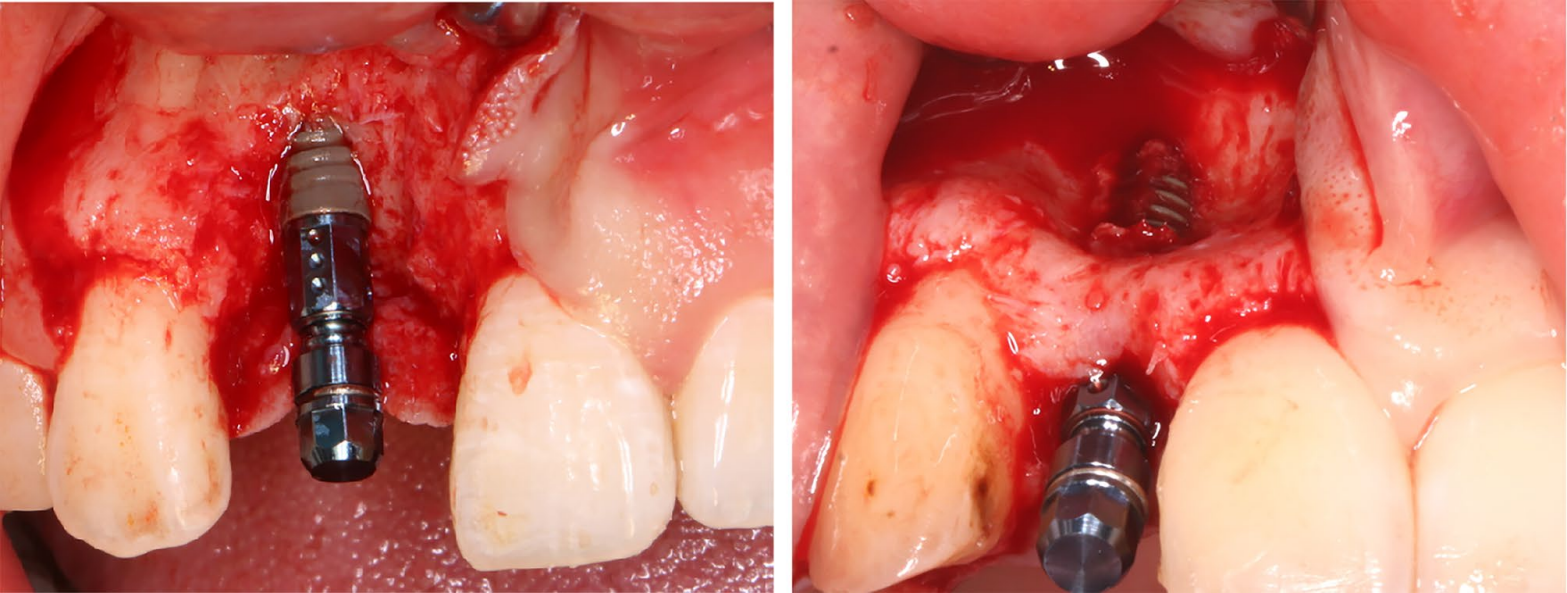
The inserted implant and the deficient alveolar crest

All surgical procedures were carried out by one experienced surgeon (QIN) under aseptic conditions. The patients were rinsed twice with 0.08% chlorhexidine gluconate gargle for 1 min for intraoral disinfection. Local infiltration anaesthesia using 4% articaine with adrenaline (1:100000) was injected throughout the surgical area. A median alveolar ridge incision and one-sided vertical releasing incision, which was at least one tooth beyond the implant site, were made by a No. 15c blade to raise a full-thickness mucoperiosteal flap. The implant sites were prepared as recommended by the manufacturer, and appropriately sized implants were placed in a prosthetically desirable position. The implant system used in the study was Straumann (Bone Level®, Straumann® Institute, Basel, Switzerland). Due to the presence of a horizontal bone defect, intraoperatively, we observed dehiscence or fenestration of the lateral labial bone, thereby exposing the implant (Fig. 1). A horizontal periosteal reduction incision was made over the elevated full-thickness flap, and the soft tissue flap was fully released and advanced to achieve tension-free closure of the wound. Bone graft sites were cleaned of any soft tissue debris, and the bone cortex was fenestrated at multiple sites using small ball drills to ensure migration of osteoprogenitor cells and neovascularization. Then, Group 1 and Group 2 underwent the following GBR procedures.

**Fig. 2:**
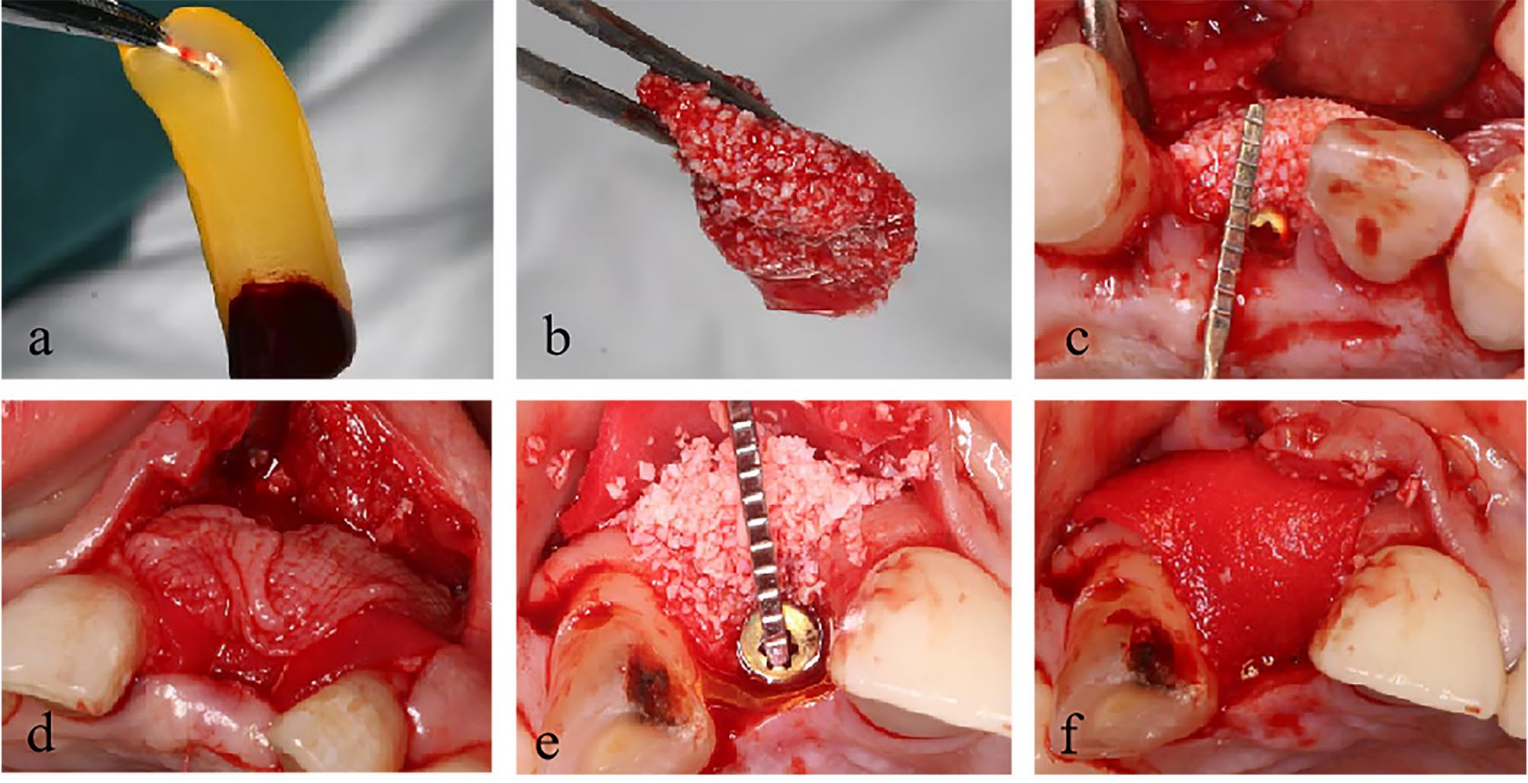
CGF (**a**), Sticky bone (**b**), Sticky bone was placed in the bone defect area and covered with collagen membrane and CGF membrane in Group 1 (**c, d**), Bone powder particles mixed with saline were placed in the bone defect area and covered with collagen membrane in Group 2 (**e, f**)

Group 1: Nine millilitres of venous blood was obtained from the patient’s forearm and collected in a sterile vacuum tube (Greiner Bio-One, GmbH, Kremsmunster, Austria) without any anticoagulant. Then, the tubes were immediately centrifuged (Medifuge, Silfradentsrl, Italy) and subjected to the following program: 30 s of acceleration, 2700 rpm for 2 min, 2400 rpm for 4 min, 2700 rpm for 4 min, 3000 rpm for 3 min, and 36 s of deceleration to stop. After this, there were three layers in the tube: the poor platelet plasma in the upper layer, the fibrin-rich gel with aggregated platelets, the CGFs in the middle layer, and the red blood cells (RBCs) in the bottom layer. The surgery requires the yellow CGF gelatine (Fig. 2a) in the middle layer and the junction with the bottom layer, which is pressed into a membrane and set aside.

Ten millilitres of venous blood was obtained from the patient and collected in a noncoated test tube, which was centrifuged at 2400–2700 rpm for 2–12 min to obtain autologous fibrin glue (AFG). The tube contained 2 different layers: the upper layer consisted of the AFG, and the RBCs in the bottom layer were discarded. The AFGs were collected in a 5 ml disposable sterile syringe and mixed with Bio-Oss® (Geistlich, Switzerland) in a sterile container. Then, CGF exudates were added to promote polymerization to produce red-coloured sticky bone (Fig. 2b). The sticky bone was moulded to fit the shape of the bone defect and inserted into the defect area to restore the full contour of the alveolar crest and ensure adequate bone volume on the labial side of the implant. The thickness of the bone graft material at the alveolar crest was measured with a probe and imaged (Fig. 2c). Finally, the sticky bone was fully covered by a collagen membrane that had been cut to fit the size and shape of the defect area. The CGF membrane was then placed over the collagen membrane and secured with a suture. (Fig. 2d).

Group 2: After full release of the soft tissue, the same bone graft mixed with physiological water was placed into the defect area with the guidance of the adjacent teeth on both sides and the contour of the alveolar ridge. The thickness of the bone graft material was measured and recorded in the same way (Fig. 2e). Similarly, the bone graft was covered with a collagen membrane fixed with a suture (Fig. 2f).

All patients were treated with the same resorbable collagen membrane (Bio-Gide™, Geistlich, Switzerland).

The wound was closed in a tension-free manner with horizontal mattress sutures and single interrupted sutures. CBCT scans were performed immediately after the surgery.

All patients were instructed to rinse with 0.2% chlorhexidine twice a day for two weeks and to take amoxicillin and ornidazole for three days. A soft diet and basic dental hygiene protocols were also advised. The sutures were removed two weeks later.

### Study variables

The bone volume conversion rate of the alveolar ridge at 6 months following surgery served as the main outcome variable of the study. The secondary study variables included visual analogue scale (VAS) pain scores, the density of newly formed bone, and the change in thickness of the labial bone graft material before and after closure of the incision.

### Clinical assessment

Participants in both treatment groups underwent clinical evaluations. Complications, including infection, wound healing conditions, graft exposure, and loss of bone fragments, were assessed. Postoperative pain was assessed by instructing patients to complete the VAS after 1 week.

### Radiographic assessment

**Fig. 3:**
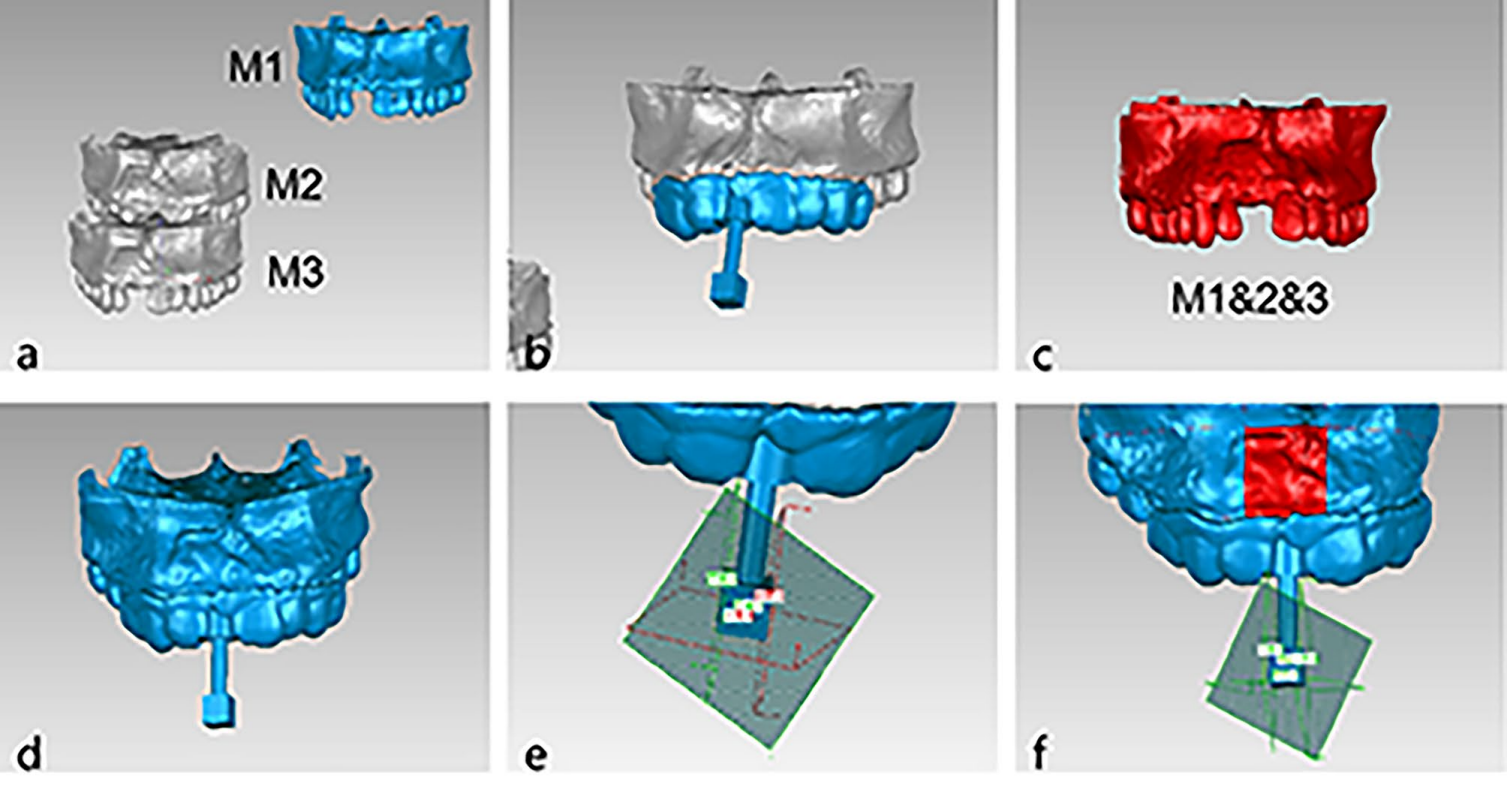
Preoperative maxillary(M1), immediate postoperative maxillary(M2) and the six-month postoperative maxillary(M3) were reconstructed (**a**), The measurement marker guide were designed according to M1 (**b**), M2 and M3 were aligned to M1 (**c**), The measurement markers were re-imported (**d**), The limiting planes were established (**e**), Three target areas were cropped and V1, V2, V3 were obtained from the software (**f**)

CBCT scans were performed on each patient before surgery, right after surgery, and 6 months postoperatively using the same projection conditions to evaluate volumetric changes at the augmentation site. CBCT data were recorded in Digital Imaging and Communications in Medicine (DICOM) file format and transferred to Mimics Research 19.0 software, where the maxillary alveolar ridge and dentition can be reconstructed in 3D (Fig. 3a).

First, we measured the labial graft thickness at the midline of the implant on CBCT images in the immediate postoperative period and compared it with the intraoperatively measured graft thickness to obtain the change in thickness after closure of the incision. Then, we selected areas of new bone on CBCT images at six months postoperatively and measured their grey values to reflect bone density.

Preoperatively, we designed a measurement marker guide in Geomagic Studio 2014 based on the preoperative maxillary model (Fig. 3b). The planes of the measurement marker corresponded to the approximate location and range of the area of bone augmentation. The purpose of the measurement marker guides was to provide several reference planes, thus ensuring that the target area remained consistent at different stages.

The three maxillary 3D image datasets were imported into Geomagic Studio 2014 software in the STL format and aligned with the dental information (Fig. 3c). The preoperatively designed measurement markers were imported, according to which the four limiting planes: the mesial, distal, palatine, and root planes, were established (Fig. 3d, e). The same target area was cropped on the three maxillary models by the plane cropping function of the software (Fig. 3f). The volume of the target areas could be obtained directly from the analysis and calculation functions of the software. The preoperative volume was calculated as V1, the immediate postoperative volume was calculated as V2, and the six-month postoperative volume was calculated as V3. The calculation (V2-V1) represents the ideal volume of bone augmentation, and the calculation (V3-V1) represents the actual volume of bone augmentation. We defined the percentage of actual bone augmentation volume to ideal bone augmentation volume as the bone volume conversion rate. Then, we calculated the bone volume conversion rate according to the following formula:

Bone volume conversion rate=(V3-V1)/(V2-V1) * 100%.

### Statistical analysis

Statistical analysis was carried out with the IBM SPSS Statistics 22.0 program (IBM Company, Armonk, NY, USA). Numerical variables are summarized by their means and standard deviations, and categorical variables are described using frequencies and percentages. The Shapiro‒Wilk test was used to evaluate the normality of the quantitative variables. Age and presurgical bone width were compared using the independent samples *t* test, and sex and implant site distributions among the groups were compared by the Fisher chi-square test. To analyse differences in quantitative variables (bone volume conversion rates, changes in graft thickness, bone density, and VAS scores) between the two groups, independent samples *t* tests were performed. For every analysis, the level of statistical significance was determined at *P*<0.05.

## Results

Overall, 28 patients were included in this study, 14 of whom were allocated into the test group (8 females and 6 males; aged 18 to 49 years; mean age 30.71 years ± 10.84 years) and 14 of whom were allocated into the control group (5 females and 9 males; aged 20 to 51 years; mean age 37.93 years ± 10.14 years). The differences in age and sex distribution were not statistically significant between the two groups (*P*=0.080 and *P*=0.449; Table 1).

There were no cases of unfavourable perioperative or postoperative outcomes during the recovery period. After 6 months, all the implants had osseointegrated, and no indications of inflammation or implant-related failure were found. The diameters of the implants placed in the incisor and canine sites were 3.3 mm and 4.1 mm, respectively. The presurgical bone width and implant site distribution, as well as the implant diameter, did not significantly differ between the two groups (*P*>0.05; Table 1). Bone dehiscence or fenestration occurred during implant drilling in the majority of patients due to the presence of horizontal bone defects, but there was no significant difference between the two groups (*P*>0.05; Table 1).

**Table 1:** Descriptive statistics of the included patients

		Group1	Group2	*P* value
Age (Mean $\pm$SD)		30.71$\pm$10.84	37.93$\pm$10.14	0.080
Gender	Male	6	9	0.449
	Female	8	5	
Augmentation sites	Central incisor	6	5	0.763
	Lateral incisor	6	8	
	Canine	2	1	
Implant diameters	3.3 mm	12	13	> 0.05
	4.1 mm	2	1	
Presurgical bone width		5.53$\pm$0.83	5.83$\pm$0.82	0.341
Dehiscence or fenestration	Yes	11	9	0.678
	No	3	5	

Assessment of the bone volume conversion rate.

**Fig. 4:**
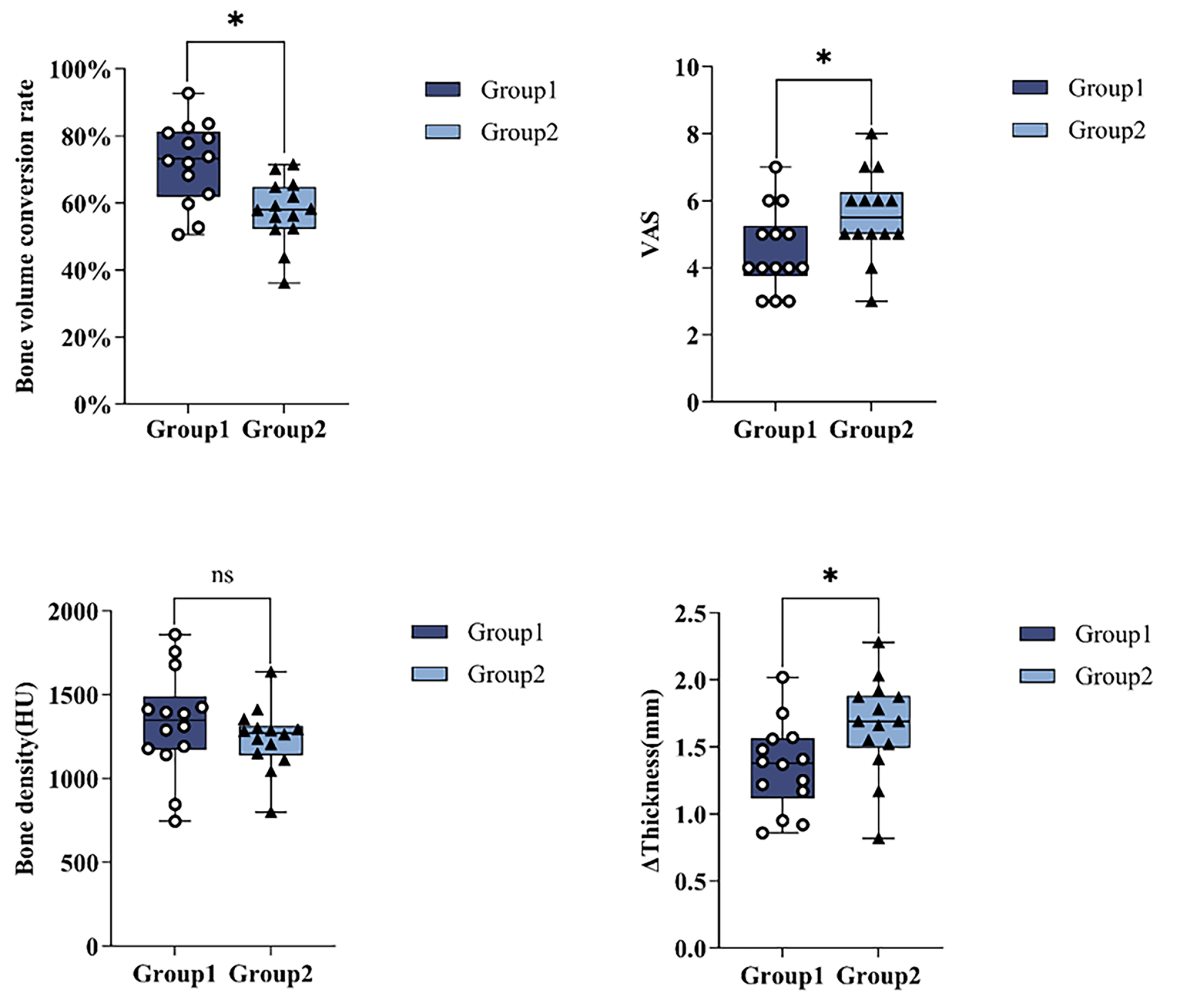
Comparison of the results of the study variables between Group1 and Group2

Sticky bone combined with CGF was used for bone augmentation, and the bone volume conversion rate was 72.09$\pm$12.18% after six months of healing in Group 1, while in Group 2, Bio-Oss mixed with saline was used for bone augmentation, and the bone volume conversion rate was 57.47$\pm$9.62% after six months. After healing, the mean bone volume conversion rates in Group 1 were significantly greater than those in Group 2 (*P*=0.002; Table 2; Fig. 4).

### Assessment of the VAS scores

A statistically significant difference was observed in the VAS score at 7 days after surgery, with a mean value of 4.50$\pm$1.22 in Group 1 and 5.57$\pm$1.28 in Group 2 (*P*=0.032; Table 2; Fig. 4).

Assessment of the density of newly formed bone.

At six months following surgery, CBCT imaging revealed that Group 1 had a greater density of newly produced bone (1330.27$\pm$311.50 HU) than did Group 2 (1240.47$\pm$189.93 HU). However, this difference was not statistically significant (*P*=0.366; Table 2; Fig. 4).

### Assessment of changes in the thickness of the labial bone graft material

The change in the thickness of the bone graft material on the labial side of the implant in Group 1 measured before and after incision closure was 1.35$\pm$0.32 mm, while that in Group 2 was 1.66$\pm$0.37 mm. The displacement of the bone graft material at the apex of the alveolar ridge due to incision closure was significantly greater in Group 2 than in Group 1 (*P*=0.025; Table 2; Fig. 4).

**Table 2:** Comparison of the results of the study variables between Group1 and Group2

Variables	Group1 (Mean $\pm$SD)	Group2 (Mean $\pm$SD)	*P* value
Bone volume conversion rate	72.09$\pm$12.18%	57.47$\pm$9.62%	0.002*
VAS	4.50$\pm$1.22	5.57$\pm$1.28	0.032*
Bone density	1330.27$\pm$311.50	1240.47$\pm$189.93	0.366
ΔThickness	1.35$\pm$0.32	1.66$\pm$0.37	0.025*

ΔThickness, the change in thickness of labial bone graft material

* Statistically significant

## Discussion

The purpose of this study was to compare the imaging volumetric changes in the target area after applying different surgical approaches for horizontal bone augmentation. The hypothesis of this study was that the combination of sticky bone and CGF is superior to conventional GBR in terms of the bone conversion rate, VAS score, density of newly formed bone, and change in thickness of the labial bone graft material. The results confirmed our hypothesis, indicating that the combination of sticky bone and CGF has a good effect on horizontal bone augmentation and can alleviate postoperative pain in patients.

Ensuring ideal aesthetic results and long-term stability is a challenge for implant therapy. Adequate labial bone volume plays a significant role in preventing peri-implant soft and hard tissue recession [22, 23]. For the consistent success of implant restorations, the presence of more than 2 mm of graft thickness on the labial side is recommended [5, 6].

There are 2 types of GBR associated with dental implants. One is concurrent with the implant, and the other precedes the implant. The size and morphology of the bone defect are the key factors in determining which method to use. In this study, we used the classification of Tolstunov et al. [21] from 2014 and selected type II, type III, and type VI alveolar ridge horizontal bone defects (i.e., alveolar ridge width ≥ 4 mm), which are suitable for implant-concurrent GBR. Good initial stability was achieved in all cases.

In the current study, the GBR bone augmentation procedure was carried out concurrently with implantation. Sticky bone combined with CGF was used in the test group, while Bio-Oss mixed with saline was used in the control group. A total of 28 patients with horizontal ridge defects in the maxillary anterior teeth were included and randomly divided into two groups. CBCT scans were performed at three different times: preoperatively, immediately postoperatively, and at 6 months, and the mean bone volume conversion rate was calculated.

The results of this study show that the bone volume conversion rate of GBR surgery using sticky bone combined with CGF is greater than that of conventional GBR surgery using Bio-Oss mixed with saline, which means that more of the bone grafts placed during the surgery were successfully converted to the desired labial bone and reflects the fact that the application of sticky bone combined with CGF may reduce the resorption of bone graft material during healing.

To the best of our knowledge, this is the first study to assess the bone augmentation effect of sticky bone in combination with CGF membranes by calculating the bone volume conversion rate inside the target area; therefore, this study cannot be directly compared with any other study. This measurement method applies a measurement marker guide to limit the target area, measure the volume, and calculate the conversion rate, which is the ratio of the actual bone gain volume to the expected bone gain volume, excluding the effect of differences in the initial alveolar ridge volume of the study subjects, which can better indicate the difference compared to a linear measurement.

Nevertheless, a study by Işık, G. et al. reported that bone resorption rates at bone gain sites using Bio-Oss in combination with platelet concentrates were lower than those at sites using Bio-Oss alone [24]. This is consistent with the findings of the current study. Although Bio-Oss is currently a very successful bone substitute material with a reliable osteogenic effect, it is completely free of any organic component and therefore lacks osteoinductive capability [25]. As a third-generation platelet concentrate product, CGF, on the other hand, has a high concentration of various growth factors, including vascular endothelial growth factor (VEGF), platelet-derived growth factor (PDGF), insulin-like growth factor (IGF), epidermal growth factor (EGF), fibroblast growth factor (FGF), transforming growth factor-β (TGF-β) and bone morphogenetic proteins (BMPs). BMP can either mediate osteogenesis on its own or, when mixed with other bone growth factors, promote the development and calcification of the bone matrix [26, 27]. TGF-β is a key regulator of the formation and remodelling of bone. It can stimulate the regeneration of alveolar bone and control inflammation by stimulating the synthesis of fibrous connective tissues and local vascular proliferation. Furthermore, several studies have demonstrated that CGF can promote bone regeneration [28]. Ji-Min et al. [29] applied CGF to maxillary sinus bone augmentation in the absence of a bone graft substitute and confirmed the ability of CGF to promote bone healing and induce new bone formation. Fang et al. [30] compared the effect of Bio-Oss® in combination with CGF or bone marrow-derived mesenchymal stem cells in canine sinus grafting and concluded that grafting with Bio-Oss® in combination with CGF can increase new bone formation more efficiently than using Bio-Oss® alone. Both the above studies and the present study demonstrate that sticky bone, as a mixture of Bio-Oss and CGF, has good osteoconductivity, and the porous nature of its structure can better function as a scaffold to promote neoangiogenesis and osteoblast growth in the matrix. At the same time, it can slowly release critical growth factors, thus obtaining a favourable bone augmentation effect.

The VAS score of Group 1 was considerably lower than that of Group 2, indicating that the use of CGF was successful in reducing patients’ postoperative pain and discomfort. In the investigation by Keranmu et al. [31], the group that used CGF combined with Bio-Oss for the alveolar ridge preservation of anterior teeth had significantly lower VAS scores on Day 1 than did the control group. In contrast, the study by Elayah et al. [32] revealed no statistically significant difference in VAS scores on the first day, but the scores were significantly lower than those of the control group on the third and seventh days; they also found that, with regard to facial swelling, the swelling values on the test side where the CGF was applied were significantly lower than those of the control group, especially on the first postoperative day (1.01 ± 0.57 vs. 1.55 ± 0.56) and on Day 3 (1.42 ± 0.8 vs. 2.63 ± 1.2). The degree of postoperative swelling was not specifically assessed in this study, but a reduction in swelling would also significantly reduce the patient’s postoperative pain and discomfort. A meta-analysis on CGF [33] also suggested that CGF has the potential to significantly improve healing and reduce pain within one week of oral surgery. The mechanism behind pain relief lies in the fact that the fibrin structure of CGF acts as a scaffolding material and reservoir to transfer growth factors, including PDGF, TGFb1, VEGF, and proinflammatory cytokines, which can greatly reduce pain caused by inflammatory responses [34].

Bone density measurements revealed that the bone density of Group 1 was slightly greater than that of Group 2, but the difference was not statistically significant; therefore, there was no difference in bone density between the two groups. This finding is consistent with the findings of Keranmu et al. [31], who reported no significant difference in the grey values measured at 6 months postoperatively. However, their study also revealed that the grey values of the alveolar bone in the CGF group were significantly greater than those in the control group at the third month. More in-depth studies are still needed on this topic.

The assessment of the change in thickness of the labial bone graft material confirmed that sticky bone has superior mechanical properties to Bio-Oss mixed with saline in resisting the pressure of wound closure. An in vitro study by Scarano et al. [35] showed that the mechanical resistance of composite scaffolds increased by 175% after the addition of autologous platelet fluid to the bone graft in comparison with blood mixed with the bone graft, and increased the mechanical resistance by 875% compared with physiological water mixed with the bone graft. When bone graft particles are mixed with autologous platelet concentrates to form sticky bone, an interlocking network of fibrin, platelets, and leukocytes is created, and the compression capability of the particulate material is improved. The ideal mechanical properties of sticky bone make the graft material more stable and prevent the bone powder particles from shifting and leaking, thus better maintaining the contour of the reconstructed alveolar ridge.

In this study, we instructed patients to take oral antibiotics to prevent postoperative infection. However, it has now been shown that autologous platelet concentrate can also be used as a carrier to release antimicrobial drugs after being prepared with antibiotic loading, thus enhancing the ability to promote healing and prevent infection [36]. This not only expands the capabilities of autologous blood products but is also a new mode of drug delivery that can compensate for the limitations of traditional systemic drug delivery, which is of great significance to the field of oral surgery.

One of the main limitations of this study is the lack of histological and histomorphometric analyses of the newly formed bone in both groups after six months of healing. In addition, this study requires long-term follow-up to observe and compare the stability of the peri-implant marginal bone levels in the two groups after the completion of restoration.

## Conclusion

Although long-term studies with larger sample sizes are still needed, it can be concluded that the application of sticky bone in combination with CGF in implant-simultaneous GBR surgeries in the presence of horizontal bone defects in the anterior region can achieve better bone augmentation results. It also helps to reduce postoperative pain and promote healing, which makes it a clinical technique worth promoting.

## Data Availability

The complete data and materials described in the research article are freely available from the corresponding author on reasonable request.

## Abbreviations

GBR: Guided bone regeneration
CGF: Concentrate growth factors
PRP: Platelet-rich plasma
PRF: Platelet-rich fibrin
AFG: Autologous fibrin glue
VAS: Visual analog scale
VEGF: Vascular endothelial growth factor
PDGF: Platelet-derived growth factor
IGF: Insulin-like growth factor
EGF: Epidermal growth factor
FGF: Fibroblast growth factor
TGF-β: Metastatic growth factors-β
BMPs: Bone morphogenetic proteins

## References

[1] Antonelli A, Bennardo F, Giudice A (2024). Breakthroughs in oral and maxillofacial surgery. J Clin Med.

[2] Heboyan A, Bennardo F (2023). New biomaterials for modern dentistry. BMC Oral Health.

[3] Benic GI, Hammerle CH (2014). Horizontal bone augmentation by means of guided bone regeneration. Periodontol 2000.

[4] Somoza-Martin JM (2021). A new morphologic classification of the alveolar ridge after distraction osteogenesis in human patients. A 17 years retrospective case series study. Med Oral Patol Oral Cir Bucal.

[5] Merheb J, Quirynen M, Teughels W (2014). Critical buccal bone dimensions along implants. Periodontol 2000.

[6] Monje A (2023). Influence of buccal bone wall thickness on the peri-implant hard and soft tissue dimensional changes: a systematic review. Clin Oral Implants Res.

[7] Wessing B, Lettner S, Zechner W (2018). Guided bone regeneration with Collagen Membranes and particulate graft materials: a systematic review and Meta-analysis. Int J Oral Maxillofac Implants.

[8] Chiapasco M, Zaniboni M, Boisco M (2006). Augmentation procedures for the rehabilitation of deficient edentulous ridges with oral implants. Clin Oral Implants Res.

[9] Chiapasco M, Casentini P (2018). Horizontal bone-augmentation procedures in implant dentistry: prosthetically guided regeneration. Periodontol 2000.

[10] Donos MRN (2010). Guided bone regeneration: biological principle and therapeutic applications. Clin Oral Implants Res.

[11] Elnayef B (2018). The fate of lateral Ridge Augmentation: a systematic review and Meta-analysis. Int J Oral Maxillofac Implants.

[12] Gultekin BA (2016). Comparison of bone resorption rates after Intraoral Block Bone and guided bone regeneration augmentation for the Reconstruction of horizontally deficient Maxillary Alveolar ridges. Biomed Res Int.

[13] Kim K (2023). Guided bone regeneration using barrier membrane in Dental Applications. ACS Biomater Sci Eng.

[14] Urban IA, Monje A (2019). Guided bone regeneration in Alveolar Bone Reconstruction. Oral Maxillofac Surg Clin North Am.

[15] Lee HM (2020). Tensile strength, growth factor content and proliferation activities for two platelet concentrates of platelet-rich fibrin and concentrated growth factor. J Dent Sci.

[16] Upadhayaya V, Arora A, Goyal A. Bioactive platelet aggregates: prp, Prgf, Prf, Cgf and Sticky Bone. IOSR J Dent Med Sci, 2017(05).

[17] Sohn DS, Huang B, Kim J, Park WE, Park CC. Utilization of autologous concentrated growth factors (CGF) enriched bone graft matrix (sticky bone) and CGF-enriched fibrin membrane in implant dentistry. J Implant Adv Clin Dent. 2015;7(10):11–29.

[18] Barbu HM et al. Guided bone regeneration with concentrated growth factor enriched bone graft matrix (sticky bone) vs. bone-Shell technique in Horizontal Ridge Augmentation: a retrospective study. Multidisciplinary Digital Publishing Institute, 2021(17). 10.3390/jcm10173953 PMC8432031 34501399

[19] Tony JB et al. CBCT Evaluation of Sticky Bone in Horizontal Ridge Augmentation with and without collagen Membrane-A randomized parallel arm clinical trial. J Funct Biomater, 2022. 13(4). 10.3390/jfb13040194 PMC9590014 36278663

[20] Alper GB (2016). Three-Dimensional Assessment of Volumetric Changes in Sinuses augmented with two different bone substitutes. Biomed Res Int.

[21] Tolstunov L (2014). Classification of the alveolar ridge width: implant-driven treatment considerations for the horizontally deficient alveolar ridges. J Oral Implantol.

[22] Chrcanovic BR (2017). Bone Quality and Quantity and Dental Implant failure: a systematic review and Meta-analysis. Int J Prosthodont.

[23] Hammerle C, Tarnow D (2018). The etiology of hard- and soft-tissue deficiencies at dental implants: a narrative review. J Clin Periodontol.

[24] Işık G (2021). Guided bone regeneration simultaneous with implant placement using bovine-derived xenograft with and without liquid platelet-rich fibrin: a randomized controlled clinical trial. Clin Oral Invest.

[25] Ferraz MP. Bone grafts in Dental Medicine: an overview of autografts, allografts and Synthetic materials. Mater (Basel), 2023. 16(11). 10.3390/ma16114117 PMC10254799 37297251

[26] Palermo A et al. Use of CGF in oral and Implant surgery: from Laboratory evidence to clinical evaluation. Int J Mol Sci, 2022. 23(23). 10.3390/ijms232315164 PMC9736623 36499489

[27] Sohn DS (2011). Bone regeneration in the maxillary sinus using an autologous fibrin-rich block with concentrated growth factors alone. Implant Dent.

[28] Chen J (2021). Considerations for clinical use of concentrated growth factor in Maxillofacial Regenerative Medicine. J Craniofac Surg.

[29] Ji-Min et al. Flapless transcrestal sinus augmentation using hydrodynamic piezoelectric internal sinus elevation with autologous concentrated growth factors alone. Implant Dent, 2014. 10.1097/ID.0000000000000053 24637529

[30] Fang, et al. A comparative study of the effect of Bio-Oss® in combination with concentrated growth factors or bone marrow-derived mesenchymal stem cells in canine sinus grafting. Journal of Oral Pathology & Medicine; 2016. 10.1111/jop.12507 27682609

[31] Keranmu D (2022). Clinical application of concentrate growth factors combined with bone substitute in alveolar ridge preservation of anterior teeth. BMC Oral Health.

[32] Elayah SA (2022). Effect of concentrated growth factor (CGF) on postoperative sequel of completely impacted lower third molar extraction: a randomized controlled clinical study. BMC Oral Health.

[33] Chen L (2023). Efficacy of concentrated growth factor (CGF) in the surgical treatment of oral diseases: a systematic review and meta-analysis. BMC Oral Health.

[34] Sun S (2023). A novel concentrated growth factor (CGF) and bio-oss based strategy for second molar protection after impacted mandibular third molar extraction: a randomized controlled clinical study. BMC Oral Health.

[35] Scarano A et al. Three-Dimensional Architecture and Mechanical properties of bovine bone mixed with autologous platelet liquid, blood, or physiological water: an in Vitro Study. Int J Mol Sci, 2018. 19(4). 10.3390/ijms19041230 PMC5979420 29670035

[36] Bennardo F (2023). Can platelet-rich fibrin act as a natural carrier for antibiotics delivery? A proof-of-concept study for oral surgical procedures. BMC Oral Health.

